# Repercussions of absolute and time-rated BMI “yo-yo” fluctuations on cardiovascular stress-related morbidities within the vascular-metabolic CUN cohort

**DOI:** 10.3389/fendo.2022.1087554

**Published:** 2023-01-09

**Authors:** Laura Sánchez-Iñigo, D. Navarro-González, D. Martinez-Urbistondo, J. C. Pastrana, A. Fernandez-Montero, J. A. Martinez

**Affiliations:** ^1^ Department of Primary Health Care of Osasunbidea, Pamplona, Spain; ^2^ Internal Medicine Department, Clínica Universidad de Navarra, Madrid, Spain; ^3^ Department of Occupational Medicine, Preventive Medicine and Public Health, University of Navarra, Pamplona, Spain; ^4^ Health Research Institute of Navarra (IdiSNA), Pamplona, Spain; ^5^ Department Physiology and Nutrition, University of Navarra (UNAV), Pamplona, Spain; ^6^ Madrid Institutes of Advanced Studies (IMDEA) Food and Health Sciences, Madrid, Spain; ^7^ Centre of Biomedical Research in Pathophysiology of Obesity and Nutrition (CIBERObn), Madrid, Spain

**Keywords:** cardiovascular disease, weight cycling, body weight trajectories, weight change slope, variability, stress, “yo-yo” pattern

## Abstract

**Aims:**

The association between body mass index (BMI) fluctuation and BMI fluctuation rate with cardiovascular stress morbidities in a Caucasian European cohort was evaluated to ascertain the impact of weight cycling.

**Methods:**

A total of 4,312 patients of the Vascular-Metabolic CUN cohort (VMCUN cohort) were examined and followed up during 9.35 years ( ± 4.39). Cox proportional hazard ratio analyses were performed to assess the risk of developing cardiovascular stress-related diseases (CVDs) across quartiles of BMI fluctuation, measured as the average successive variability (ASV) (ASV = |BMIt0 − BMIt1| + |BMIt1 − BMIt2| + |BMIt2-BMIt3| +…+ |BMItn – 1 − BMItn|/*n* − 1), and quartiles of BMI fluctuation rate (ASV/year).

**Results:**

There were 436 incident cases of CVD-associated events involving 40,323.32 person-years of follow-up. A progressively increased risk of CVD in subjects with greater ASV levels was found. Also, a higher level of ASV/year was significantly associated with an increased risk of developing CVD stress independent of confounding factors with a value of 3.71 (95% CI: 2.71-5.07) for those in the highest quartile and 1.82 (95% CI: 1.33-2.50) for those in the third quartile.

**Conclusions:**

The BMI fluctuation rate seems to be a better predictor than BMI fluctuation of the potential development of cardiovascular stress morbidities. The time-rated weight fluctuations are apparently more determinant in increasing the risk of a CVD than the weight fluctuation itself, which is remarkable in subjects under “yo-yo” weight patterns for precision medicine.

## Introduction

Obesity is a well-established risk factor for cardiovascular disease (CVD) incidence of stress-related clinical manifestations (1). Most cardiovascular prevention guidelines recommend weight stability maintenance and weight loss in those subjects with obesity, but many patients fail to do so, resulting in weight cycling (2, 3). Stress and anxiety exposure can also have a significant long-term impact on the alignment to gain and maintain weight due to inadequate compensatory mechanisms or maladaptive processes, which may lead to rapid and frequent weight “yo-yo” fluctuations (4–6). Actually, studies on overweight and obese individuals reported that only 20% of participants were able to maintain long-term weight loss (7).

Body-weight fluctuation, which is also named body-weight variability or weight cycling, refers to repeated weight loss and successive regain (8). Several years ago, the first evidence that body-weight variability could be related to a high risk of CVD arose from the Framingham cohort (9). Later, several studies evaluated the association of body-weight variability with CVD outcomes and deaths among the general population and patients with type 2 diabetes (T2D) or previous CVD (10–17). However, while some epidemiological studies have shown that body-weight fluctuations may be associated with metabolic disorders and complications, resulting in negative health consequences (9, 18, 19), other cohort studies have failed to confirm these findings or even get opposite results concerning mild-weight fluctuations (13, 14, 20–22). These investigations have typically focused on the variability of weight, an imprecise measure of adiposity, and did not assess the variability of markers of overall obesity such as body mass index (BMI), which captures both fat and lean mass composition (11), and neither assessed the time-rated velocity of these fluctuations and variability (11, 23). Moreover, there is not a single definition or measurement concerning weight cycling matters (8), and to our knowledge, the involvement of intensive BMI fluctuations in affecting the association with cardiovascular stress-related morbidities has not been previously examined. Therefore, the aim of the present study concerning weight cycling was to evaluate the association between BMI fluctuation and BMI fluctuation rate with incident CVD in a Caucasian European cohort.

## Methods

### Subjects

**Figure 1:**
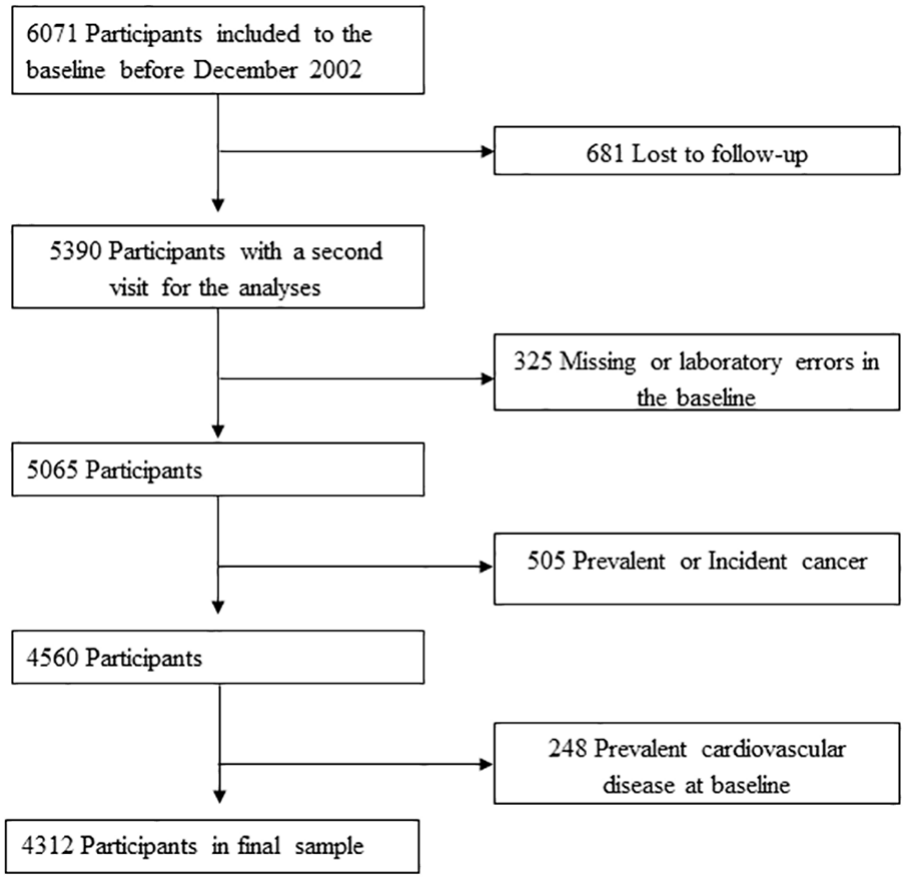
Flowchart of study participants drawn from the Vascular-Metabolic CUN cohort between 1997 to 2012.

The Vascular-Metabolic CUN cohort (VMCUN cohort) is a population-based, epidemiological study designed to examine the incidence of cardiovascular and metabolic diseases including T2D, hypertension, obesity, stroke, or coronary heart disease in a large European population. We collected data from the medical history of first-time attendee outpatients to the Internal Medicine Department at the Clínica Universidad de Navarra (CUN) from 1 February 1997 to 31 December 2002, and they were subsequently followed up until 31 December 2012. Thus, the cohort has been described elsewhere (24). Briefly, the exclusion criteria were age younger than 18 or older than 90 years old, history of type 1 diabetes or latent autoimmune diabetes in adults, cancer in the palliative phase, familial hypertriglyceridemia, extreme BMI (>45 kg/m^2^), bariatric surgery, or hypercoagulability. Of the initial 6,071 participants included, 681 without one valid follow-up weight measurement were lost, and 325 were omitted because of erroneous and missing required laboratory values. Additionally, we excluded for the analyses 505 participants with prevalent or incident cancer during follow-up because of likely interactions with BMI fluctuations. Finally, 248 participants with baseline CVD were removed. These requisites left 4,312 participants available for the final baseline analyses (**Figure 1**). The research was conducted according to the standards of the Declaration of Helsinki on medical research (25) and was approved by the Ethics Committee of the Universidad de Navarra (30/2015).

### Measurements

Data regarding medical history, health-related behaviors, and blood biochemical measurements were retrieved at each patient’s visit. Only patients with at least one valid follow-up weight measurement were included in the BMI fluctuation analysis.

Health-related behaviors including cigarette smoking (none, former smoker, or current smoker), daily alcohol intake (yes/no), and lifestyle pattern (physically active/sedentary behavior) were obtained by physicians at the consultation. Prior to the measurement of blood pressure (BP), subjects waited for 5 min in a seated position. The BP on the indistinctly right or left arm was measured twice, and the average value was recorded following universal standardized procedures (26). Hypertension was defined on the basis of the World Health Organization-International Society of Hypertension Guidelines (26) as ≥140 (systolic BP)/90 (diastolic BP) mmHg or when the subjects reported the use of antihypertensive medication. Blood samples were drawn after an 8-h fast and analyzed in a central laboratory with a Hitachi 711 Chemistry Analyzer under validated strict quality controls. Fasting plasma glucose (FPG) was measured by the hexokinase method, while total cholesterol (TC), high-density lipoprotein cholesterol (HDL-C), and triglycerides (TG) were measured using enzymatic colorimetric tests. Low-density lipoprotein cholesterol (LDL-C) was conventionally estimated using the Friedewald formula. The values of LDL-C were treated as missing in subjects with TG levels greater than 400 mg/dl (0.2% of the participants). The triglyceride–glucose (TyG) index was calculated as ln[fasting TG (mg/dl) × FPG (mg/dl)/2] as previously reported (27). TG/HDL-C ratio was calculated as TG divided by HDL-C (expressed in mg/dl).

### Definitions of BMI fluctuations

Anthropometric measurements (weight, height, and BMI) were performed by a trained nurse according to standardized operation procedures (24). Thus, weight was quantified with subjects wearing light clothing and to the nearest 0.1 kg; height was measured without shoes to the nearest 0.1 cm. BMI was calculated as the body mass divided by the square of the body height and expressed in units of kg/m^2^. The participants were categorized as normal weight, overweight, or obese by the commonly accepted BMI ranges for clinical analyses (28).

BMI fluctuation as a marker of adiposity cycling was defined as the intraindividual variability in BMI between visits (9). Various measures of variability were used, including the average successive variability (ASV), which was defined as the absolute difference between successive values (10, 11, 14). The ASV of BMI was calculated with the BMI at each visit during the follow-up (BMItn) and using the following formula: |BMIt0 − BMIt1| + |BMIt1 − BMIt2| + |BMIt2 − BMIt3| +…+ |BMItn − 1 − BMItn|/*n* − 1, as described elsewhere (14). The BMI fluctuation rate, as the absolute difference between successive values of BMI per year: ASV/years of follow-up, was also estimated. The ASV of BMI by including the BMI values from baseline to just the event was captured.

### Definition of CVD and assessment of stress risk factors

Cardiovascular disease was defined according to the International Classification of Diseases, Tenth Revision (ICD-10), as published elsewhere (29). The code list covers diseases from three groups: coronary heart disease (CHD), codes from I20 to I25 (angina pectoris, acute myocardial infarction and subsequent complications, and chronic ischemic heart disease); cerebrovascular disease, codes from I63 to I66 (cerebral infarction, stroke, occlusion, and stenosis of precerebral arteries and cerebral arteries); and peripheral arterial disease, codes I73.9 and I74 (intermittent claudication, arterial embolism, and thrombosis). Metabolic stress-related determinants such as smoking, alcohol intake, and lifestyle pattern were addressed with validated tools (24).

### Statistical analyses

The mean of follow-up was 9.35 ± 4.4 years, with a median number of 3 visits per patient (range 2 to 8 visits) and a median time gap of 2 years between each clinical visit in order to follow “yo-yo” cycling in this population. Continuous variables were expressed as the mean ± standard deviation (SD). Categorical variables were presented as percentages (%). The Student’s *t*-test, one-way ANOVA, or *χ* ^2^ test was used to compare the characteristics of the BMI fluctuation rate quartiles as appropriate. A general linear model was used to fit the median of the quartiles as a continuous variable to estimate the trend of variables across quartiles. We used the multiple imputation procedure in STATA version 13 (mi command) to impute the missing data of the variables cigarette smoking (16.5% missing values), daily alcohol intake (25.2% missing values), and lifestyle pattern (29.9% missing values). Twenty imputed datasets were created to reduce sampling variability from the imputation process. The variables included in the imputation procedure were baseline age, sex, BMI, T2D hypertension, TyG index, FPG, TG, TC, HDL-C, ASV, ASV/years, and the outcome of cardiovascular disease following standard operation procedures (30). A run length of 100 iterations was used between data sets. All variables included before imputation had a normal distribution.

Cox proportional hazard analysis was conducted to estimate the hazard ratio (HR) and the 95% CI of cardiovascular disease in each quartile of BMI fluctuation and BMI fluctuation rate. The lowest quartile of risk was determined as the reference category, and repeated-measures ANOVA was used to assess changes over time. We fitted three models: a crude (univariate) model and two Cox regression multivariate-adjusted models—a) controlling for age (continuous) and sex (as an interaction factor) and b) additionally adjusted for baseline BMI (continuous), cigarette smoking (never, current, and former smokers), daily alcohol intake (yes/no), lifestyle pattern (physically active/sedentary behavior), hypertension (yes/no), T2D (yes/no), LDL-C (continuous) and HDL-C (continuous), TG (continuous), antiaggregation therapy (yes/no), number of visits during follow-up (continuous), and the direction of the change in BMI, as a result of the BMI at the end of follow-up minus baseline BMI (neutral/positive/negative).

The analyses were stratified by baseline BMI, sex, and age groups (<50, 50-65, and >65 years old), and a Cox regression multivariate-adjusted model was conducted including the covariates in model (c), except baseline BMI, sex, or age, respectively. The interaction terms between baseline BMI/sex/age groups and the quartiles of BMI fluctuation and BMI fluctuation rate were fitted to test for differences in association.

Finally, a sensitivity analysis was performed to evaluate the robustness of the results. A logistic regression model was conducted among 3,409 participants after 5 years of follow-up, excluding any incident cases of a cardiovascular event during the 5-year period, to assess the odds ratio (OR) between CVD and the quartiles of BMI fluctuation and BMI fluctuation rate. The logistic regression was adjusted as previously described before for the Cox model.

All statistical analyses were performed with STATA version 13 (Stata Corp., College Station, TX, USA), whose manuals were applied (31). All *p*-values are two-tailed and statistical significance was set at the conventional cutoff of *p <*0.05.

## Results

Data from 1,725 women and 2,587 men with a mean ( ± SD) age at baseline of 53.7 ± 13.3 were screened. After 40,323.32 person-years of follow-up, there were 436 incident cases of CVD. Age, BMI, and the prevalence of hypertension were more likely to increase across quartiles of BMI fluctuation and BMI fluctuation rate (**Tables 1A**, **1B**). Furthermore, this trend was found associated with higher frequencies of sedentary behavior. The values for TG and TyG index increased, and the level of HDL-C decreased in proportion to both BMI fluctuation and BMI fluctuation rate quartiles (**Tables 1A**, **1B**). The proportion of women and antiaggregation therapy increases across the quartiles of BMI fluctuation (**Table 1A**).

**Table 1a:** Baseline demographics and risk factor profiles according to quartiles of body mass index (BMI) fluctuation (|kg/m^2^|) of 4,312 participants drawn from the VMCUN clinical cohort between 1997 and 2015 in Spain.

		BMI fluctuation				
	Overall	Q1 (0-0.55)	Q2 (0.56-0.91)	Q3 (0.92-1.47)	Q4 (>1.47)	*p*
*n*	4,312	1,078	1,079	1,080	1,075	
Sex (% women)	40.0	36.4-	38.3	41.0	44.5	0.001
Age (years)	53.7 ± 13.3	53.8 ± 12.5	54.2 ± 12.9	53.3 ± 13.1	53.4 ± 14.6	0.404
BMI (kg/m^2^)	26.8 ± 4.2	26.1 ± 3.6	25.9 ± 3.8	27.1 ± 4.3	27.9 ± 4.9	<0.001
Hypertension (%)	24.2	21.5	23.9	23.9	27.4	0.015
Type 2 diabetes (%)	4.9	5.1	4.5	4.4	5.9	0.376
Antiaggregation therapy (%)	10.3	8.2	10.3	11.6	11.0	0.042
Fasting plasma glucose (mg/dl)	98.9 ± 22.3	98.8 ± 25.6	98.3 ± 21.9	98.3 ± 20.0	100.1 ± 21.5	0.225
Total cholesterol (mg/dl)	224.5 ± 41.3	226.5 ± 39.2	224.3 ± 42.3	223.5 ± 40.4	223.8 ± 43.1	0.338
HDL cholesterol (mg/dl)	55.3 ± 15.0	56.0 ± 15.2	56.2 ± 15.3	54.8 ± 14.8	54.1 ± 14.4	0.003
Triglycerides (mg/dl)	100.9 ± 63.5	98.0 ± 59.0	97.9 ± 58.9	101.8 ± 62.4	105.7 ± 72.4	0.013
TyG index	8.4 ± 0.6	8.3 ± 0.6	8.3 ± 0.6	8.4 ± 0.6	8.4 ± 0.6	0.009
Smoking (%)						0.146
Current smokers	33.6	32.0	32.5	32.5	37.3	
Former smokers	18.2	18.3	17.3	18.2	18.4	
Alcohol intake (%)						0.579
Daily drinkers	47.9	49.8	48.3	46.6	47.0	
Lifestyle activity (%)						<0.001
Sedentary behavior	57.0	52.7	49.7	61.3	64.2	
Number of visits	3.5 ± 2.3	3.2 ± 2.3	4.1 ± 2.3	3.8 ± 2.2	2.9 ± 2.0	<0.001
Time between visits (years)	3.4 ± 2.5	3.3 ± 2.4	2.8 ± 1.9	3.3 ± 2.3	4.1 ± 2.9	<0.001
Body-weight change and fluctuation						
BMI fluctuation (|kg/m^2^|)	1.2 ± 1.0	0.3 ± 0.2	0.7 ± 0.1	1.2 ± 0.2	2.5 ± 1.2	<0.001
BMI fluctuation rate (|kg/m^2^/year|)	0.6 ± 1.1	0.2 ± 0.6	0.5 ± 1.3	0.7 ± 1.0	1.1 ± 1.2	<0.001

BMI fluctuation quartile data are presented as mean ± SD or % as appropriate. p <0.05 by one-way ANOVA (continuous variable) or χ^2^ test (categorical variables).

SD, standard deviation; HDL-C, high-density lipoprotein cholesterol; TyG index, triglyceride–glucose index.

**Table 1b:** Baseline demographics and risk factor profiles according to quartiles of body mass index (BMI) fluctuation rate (|(kg/m^2^)/year|) of 4,312 participants drawn from the VMCUN clinical cohort between 1997 and 2015 in Spain.

		BMI fluctuation rate			
	Q1 (0-0.2)	Q2 (0.21-0.39)	Q3 (0.4-0.71)	Q4 (>0.71)	*p*
*n*	1,078	1,078	1,078	1,078	
Sex (% women)	39.2	38.3	42.3	40.4	0.257
Age (years)	52.3 ± 12.9	53.4 ± 12.6	54.0 ± 12.9	54.9 ± 14.6	<0.001
BMI (kg/m^2^)	25.7 ± 3.5	26.4 ± 3.9	27.1 ± 4.3	27.8 ± 4.8	<0.001
Hypertension (%)	19.9	21.3	25.3	30.1	<0.001
Type 2 diabetes (%)	4.1	4.2	4.6	6.9	0.006
Antiaggregation therapy (%)	8.8	11.1	10.6	10.7	0.304
Fasting plasma glucose (mg/dl)	97.2 ± 23.0	98.3 ± 22.1	98.3 ± 18.9	101.7 ± 24.8	<0.001
Total cholesterol (mg/dl)	226.1 ± 39.8	224.0 ± 41.0	226.0 ± 41.5	222.0 ± 42.7	0.069
HDL cholesterol (mg/dl)	57.0 ± 15.3	55.8 ± 15.3	54.7 ± 14.5	53.6 ± 14.6	<0.001
Triglycerides (mg/dl)	94.0 ± 57.1	96.4 ± 55.2	105.0 ± 64.3	108.2 ± 74.5	<0.001
TyG index	8.3 ± 0.6	8.3 ± 0.6	8.4 ± 0.6	8.4 ± 0.6	<0.001
Smoking (%)					0.016
Current smokers	29.5	32.9	34.5	37.7	
Former smokers	18.0	18.8	18.2	17.7	
Alcohol intake (%)					0.478
Daily drinkers	46.8	49.9	46.5	48.4	
Lifestyle activity (%)					<0.001
Sedentary behavior	48.5	55.3	58.8	65.9	
Number of visits	2.2 ± 1.7	3.7 ± 2.2	4.3 ± 2.3	3.8 ± 2.3	<0.001
Time between visits (years)	5.1 ± 3.2	3.4 ± 2.7	2.8 ± 1.6	2.2 ± 1.2	<0.001
Body-weight change and fluctuation					
BMI fluctuation (|kg/m^2^|)	0.4 ± 0.2	0.9 ± 0.3	1.3 ± 0.5	2.2 ± 1.5	<0.001
BMI fluctuation rate (|kg/m^2^/year|)	0.1 ± 0.1	0.3 ± 0.1	0.5 ± 0.1	1.5 ± 1.8	<0.001

BMI fluctuation rate quartile data are presented as mean ± SD or % as appropriate. p <0.05 by one-way ANOVA (continuous variable) or χ^2^ test (categorical variables).

SD, standard deviation; HDL-C, high-density lipoprotein cholesterol; TyG index, triglyceride–glucose index.

We found a slightly significant (*p* < 0.05) increased risk of CVD among the quartiles of BMI fluctuation, with an HR of 1.34 (95% CI: 1.00-1.81) for those participants in the fourth quartile of BMI fluctuation (**Table 2**). However, in the multivariate-adjusted model, a higher and significant and progressive increase in the risk of CVD with BMI fluctuation rate was found in the third and fourth quartiles as compared with the bottom quartile: HR of 1.82 (95% CI: 1.33-2.50) and HR of 3.71 (95% CI: 2.71-5.07), respectively (*p* for trend <0.001) (**Table 2**).

**Table 2:** Risk of incident cardiovascular disease (CVD) according to body mass index (BMI) fluctuation (|kg/m^2^|) and BMI fluctuation rate (|kg/m^2^/year|) of 4,312 participants drawn from the Vascular-Metabolic CUN clinical cohort between 1997 and 2015.

	BMI fluctuation (|kg/m^2^|) and incident cardiovascular disease				
	Q1 (0-0.55)	Q2 (0.56-0.91)	Q3 (0.92-1.47)	Q4 (>1.47)	*p* for trend
*n*	1,078	1,079	1,080	1,075	
Number of incident cases of CVD	110	111	100	115	
Incidence (%)	10.20	10.30	9.26	10.70	
Person-years	9,318.8	10,641.3	10,875.6	9,487.5	
Incidence/1,000 person-years	11.80	10.43	9.19	12.12	
Crude	1 (ref)	0.85 (0.65-1.11)	0.78 (0.60-1.02)	1.09 (0.84-1.42)	0.292
Age- and sex-adjusted	1 (ref)	0.87 (0.67-1.13)	0.82 (0.63-1.08)	1.20 (0.92-1.56)	0.075
Multivariate adjusted model	1 (ref)	0.98 (0.75-1.28)	0.93 (0.70-1.24)	1.34 (1.00-1.81)	0.023
	BMI fluctuation rate (|(kg/m^2^)/year|) and incident cardiovascular disease				
	Q1 (0-0.2)	Q2 (0.21-0.39)	Q3 (0.4-0.7)	Q4 (>0.7)	*p* for trend
*n*	1,078	1,078	1,078	1,078	
Number of incident cases of CVD	64	81	123	168	
Incidence (%)	5.93	7.51	11.41	15.58	
Person-years	12,375.73	12,276.91	10,068.1	5,602.5	
Incidence/1,000 person-years	5.18	6.59	12.21	29.98	
Crude	1 (ref)	1.13 (0.82-1.56)	1.66 (1.22-2.26)	3.54 (2.62-4.77)	<0.001
Age- and sex-adjusted	1 (ref)	1.16 (0.84-1.59)	1.84 (1.35-2.51)	3.84 (2.84-5.20)	<0.001
Multivariate adjusted model	1 (ref)	1.16 (0.84-1.60)	1.82 (1.33-2.50)	3.71 (2.71-5.07)	<0.001

The multivariate Cox model was adjusted for age, sex, baseline BMI, cigarette smoking (never, current, and former smokers), daily alcohol intake (yes/no), lifestyle pattern (physically active/sedentary behavior), hypertension, type 2 diabetes, antiaggregation therapy, number of visits during follow-up, direction of the change in BMI (neutral/positive/negative), HDL cholesterol, LDL cholesterol, and triglycerides.

**Figure 2:**
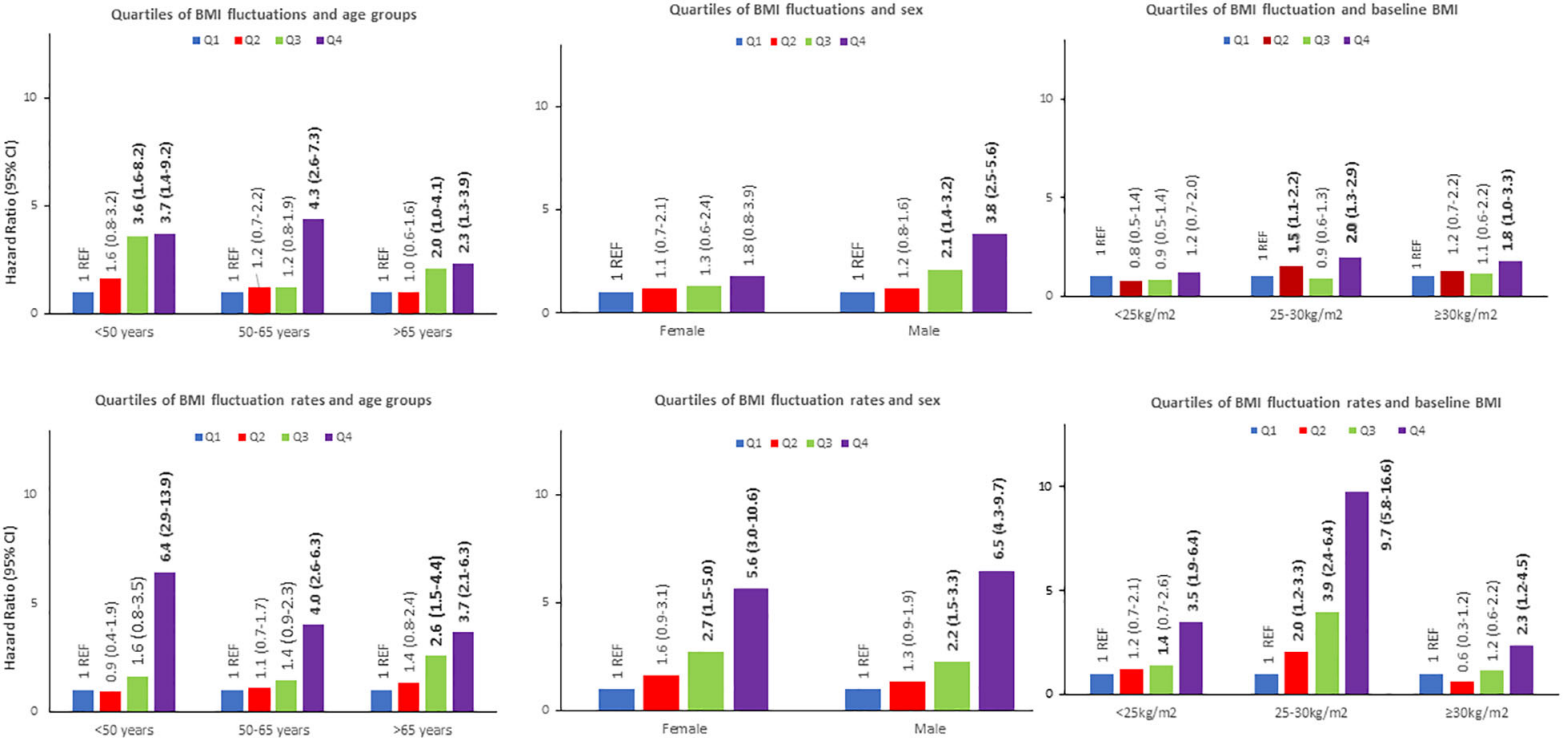
Absolute body mass index (BMI) fluctuation (|kg/m2|) and absolute BMI fluctuation rates (|kg/m2/year|) influence on the risk of developing a CVD according to age groups, sex and baseline BMI.

The stratified analysis by baseline BMI, sex, and age groups showed a heightening risk of CVD in all age subgroups, with an HR of 3.7 (95% CI: 1.4-9.2), 4.3 (95% CI: 2.6-7.3), and 2.3 (95% CI: 1.3-3.9) for the highest quartile of BMI fluctuation, of the age groups of <50, 50-65, and >65 years old, respectively (**Figure 2**). We also observed a higher risk in men among those of the third and fourth quartiles, with an HR of 2.1 (95% CI: 1.4-3.2) and 3.8 (95% CI: 2.5-5.6), respectively, and a higher risk in overweight and obese participants of the fourth quartile, with an HR of 2.0 (95% CI:1.3-2.9) and 1.8 (95% CI:1.0-3.3) in that order (**Figure 2**). Remarkably, BMI fluctuation was not associated with CVD in women and normal-weight participants (**Figure 2**), but this was just a trend.

The potential involvement of age, sex, and baseline BMI on the relation of quartiles of BMI fluctuation rate to incident CVD was also addressed (**Figure 2**). Thus, the incidence of CVD increased in proportion to the quartiles of BMI fluctuation rate in all the groups of the stratified analysis. Interestingly, the HRs for developing a CVD were 5.6 (95% CI: 3.0-10.6) and 6.5 (95% CI: 4.3-9.7) for women and men in the fourth quartile, respectively. Noteworthy, the risk of a CVD was larger in overweight/normal-weight than obese participants. Across the age subgroups, the greater risk of CVD was found in the youngest participants, subgroups age <50 and 50-65 years old, reaching an HR of 6.4 (95% CI: 2.9-13.9) and 4.0 (95% CI: 2.6-6.3) for those participants of the fourth quartile of BMI fluctuation rate, respectively (**Figure 2**).

**Figure 3:**
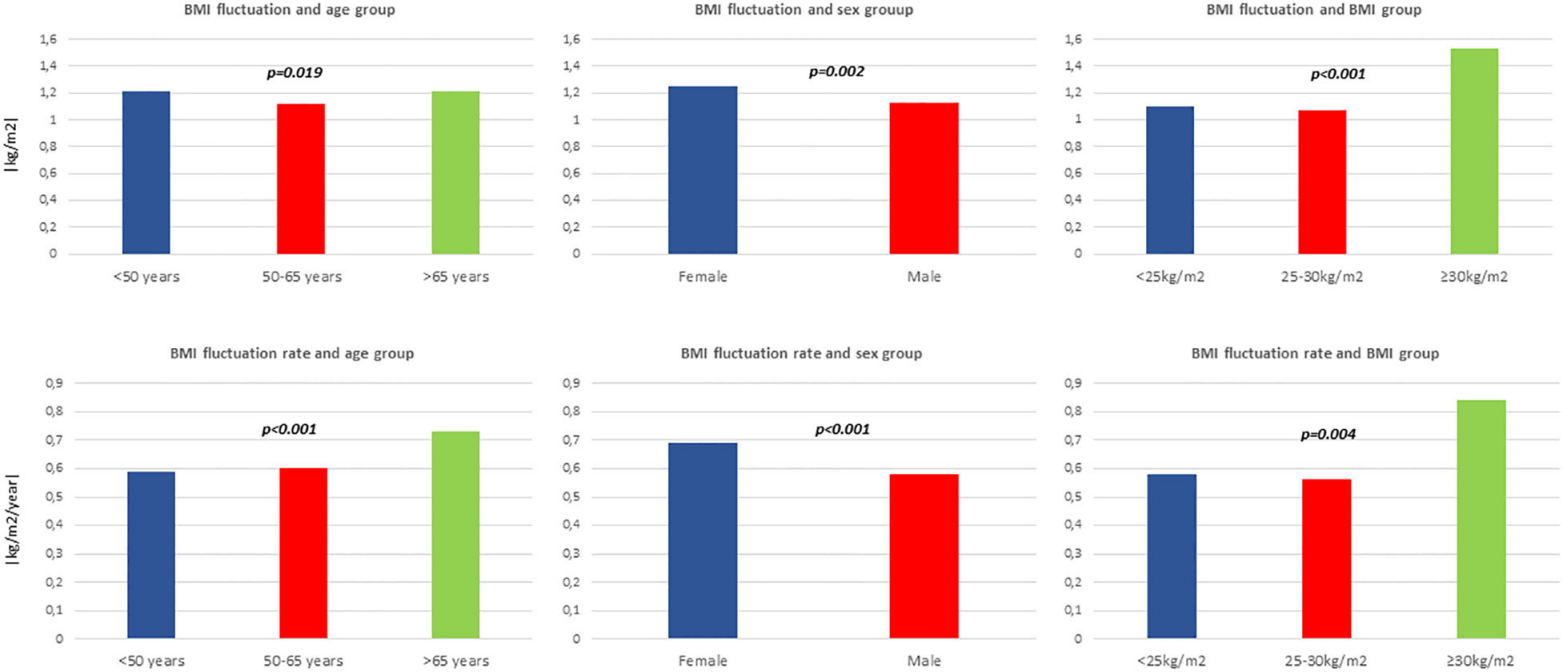
Mean body mass index (BMI) fluctuation (|kg/m2|) and mean BMI fluctuation rate (|kg/m2/year|) according to age groups, sex and baseline BMI.

The magnitude of the associations found across the quartile of BMI fluctuation rate and the outcome of a CVD was larger than that displayed by using BMI fluctuation (**Figure 2**). The differences between the mean of BMI fluctuation and BMI fluctuation rate according to baseline BMI, sex, and age subgroups were statistically significant (**Figure 3**).

The main results of the present study were consistent with a time-limited scenario of 5 years that we included in the sensitivity analysis (**Supplemental Table 1**). An association between the risk of CVD among the third and fourth quartiles of BMI fluctuation and BMI fluctuation rate was found, with a robustness OR when using the BMI fluctuation rate (OR of 1.59 and 1.95 *vs*. OR of 2.19 and 3.45, for the third and fourth quartiles, respectively) as reported (**Supplemental Table 1**).

## Discussion

This research study showed that BMI fluctuation rate is a stronger predictor than just BMI fluctuation in the development of cardiovascular-related events. This approach is apparently the first analysis specifically focused to evaluate BMI fluctuation rate and cardiovascular outcomes, despite that many investigations have found that body-weight fluctuations may be associated with metabolic disorders and complications (23).

The pathophysiological mechanisms behind this increased risk in CVD in higher BMI fluctuation are under study, and different hypotheses have been proposed (3). Studies on animals and humans revealed the involvement of weight fluctuations in adipose tissue inflammation, adipose hypertrophy, and oxidative stress, which are conditions that stimulate the development of insulin resistance (32–35) and lead to cardiovascular complications (12). This cyclical oscillation could also be associated with abdominal and visceral fat accumulation (36), negatively affecting insulin levels and causing inflammation (37, 38). Therefore, weight cycling and the “yo-yo” pattern lead to insulin resistance, adipocyte inflammation, and metabolism disorders, resulting in increased oxidative stress and endothelial dysfunction, leading to atherosclerosis (39, 40). Epigenetic pathways are in many cases adaptive mechanisms to internal situations (7). It has been widely studied that the expression of candidate genes related to obesity and cardiovascular diseases is associated with DNA methylation and histone acetylation, consequential in the changes in cellular phenotypes and cardiac function (41). Weight “yo-yo” fluctuations may enhance epigenetic adaptation to unbalanced nutrition, altering gene expression and constitution (7). In fact, several epigenetic alterations were discovered in response to weight loss interventions, and genome-wide methylation analysis of adipose tissues after 6 months of endurance training demonstrated changes in DNA methylation of 63 genes involved in obesity (42). These genes participate in metabolic pathways, cytoskeletal organization, cell adhesion, and cell signaling (42). Transcriptomic studies in patients subjected to weight cycling showed the promoted expression of genes involved in the activation of pathways related to the formation of fibrin clot, cardiomyopathy, lipids, and cell surface interaction at the vascular wall (43–46). Weight regain results in rapid adipose tissue growth and hyperplasia, with an increase of cytokines, such as leptin, which acts on the limbic system by stimulating dopamine uptake, creating a feeling of fullness (47). However, these adipokines induce the production of reactive oxygen species as a process of mitochondrial oxidative phosphorylation, engendering oxidative stress (47). The stability of weight often is not achieved, resulting in weight fluctuations and the beginning of a new weight cycling (7). Interestingly, these pathways were only affected after weight regain (43). The central nervous system with homeostatic control centers within the hypothalamus and hindbrain appears to be the key for the maintenance of body weight (7). Cortisol is involved in stress response through the sympathoadrenal system and the hypothalamic–pituitary–adrenocortical axis (48–50). These regulatory mechanisms have been critical for the survival of individuals and species but may become maladaptive when stress is intense or chronic and may bring on rapid weight fluctuations (4, 51, 52). Also, some researchers reported that patients with weight fluctuations had significantly lower general well-being, minor eating self-efficacy, and greater stress than weight maintenance patients, regardless of body weight (53). Additionally, other investigators found that individuals who reported stressful life events had significantly higher odds of having metabolic syndrome (54). On the other hand, it has been shown that patients who regained weight after losing weight, whom they called “relapsers,” had greater levels of serum leptin and insulin than weight maintainers (55). Ultimately, the accumulation of stressful life events has been associated with insulin resistance, obesity, raised triglycerides and oxidized LDL-C, and cardiovascular stress-related morbidities (4–6, 56).

Several epidemiological studies have described the association between weight fluctuations and CVD, especially in patients with T2D or a previous CVD (8, 9, 13–18). However, other cohort studies have failed to confirm these findings or even found opposite outcomes and trends (13, 14, 20–22, 57, 58). A meta-analysis, with 25 studies involving more than 400,000 participants, reported no effect in more than 60% of both total participants and events (18). The inconsistency in some results may be due to several factors, such as the heterogeneity of the measurement of weight fluctuation, baseline BMI, and age. In this context, a strong association between body-weight fluctuations and the risk of cardiovascular events among participants with coronary artery disease has been described (11). However, no data on baseline BMI were reported, which is a limitation, and rapid body-weight fluctuation should be considered in further studies concerning weight “yo-yo” outcomes (23).

Some interesting results arose from the subgroup analyses in our study. Thus, the stronger association using BMI time-rated fluctuations rather than BMI fluctuations and the risk of developing a CVD according to baseline BMI, sex, and age groups was consistent in all subgroups. In addition, we found a weaker association using BMI fluctuation, particularly in women and in the analysis stratified by baseline BMI. The highest quartile of BMI fluctuation was associated with the risk of CVD in all age subgroups, men, and overweight and obese patients. These results are consistent with other previous observations (14, 15, 18). Interestingly, the risk of a CVD was lower in the fourth quartile of the BMI fluctuation rate in obese patients, compared with the HR found for normal-weight and overweight participants. This outcome could be due to the baseline increased risk associated with obesity, the “obesity paradox” reported in previous studies: obese individuals may have developed a CVD because they were obese or because of previous “yo-yo” weight fluctuations (3, 59). Thus, these results should be examined with caution. Another possible explanation is the potential bias due to related weight loss. Weight fluctuations in obese individuals probably would have a major trend toward losing weight and reducing cardiovascular risk. This bias may serve to increase morbidity risk in the non-obese groups, creating the appearance that obesity is protective (59). Moreover, the statistically significant differences between the mean of BMI fluctuation according to baseline BMI, sex, and age subgroups shown in our study suggest that weight fluctuations should be addressed independently or stratified by these groups. However, further studies are warranted to confirm these associations.

BMI was applied to evaluate fluctuation and fluctuation rate according to a better clinical approach and to be able to compare patients with different heights (9). The gain of any kilograms should be in the context of the height of a patient. The use of BMI to examine weight fluctuations may allow us to compare different groups of patients and to open up in other populations or cohorts, despite limitations in some cases (18).

Interestingly, BMI time-rated fluctuation is not just a stronger predictor to develop a CVD, as shown in our study, but it has a clinical and practical message for the management of patients. Therefore, weight maintenance is recommended to minimize weight fluctuations and avoid rapid BMI cycling. Indeed, it appears that the speed or velocity of the fluctuation acts as a primary determinant. A key point for patients is to be aware of losing or gaining weight in short periods of time, and the accumulation of these rapid “yo-yo” weight fluctuations would increase their cardiovascular risk and complications.

The several limitations of this study also warrant consideration. We did not record lifestyle habits such as nutrition and energy intake. Although we have not adjusted for this possible confounding factor, we used other additional variables to adjust, such as BMI or cholesterol levels, which are indirectly related to nutritional habits. Future studies are needed to confirm the specific relationship between other CVD stress-related diseases and T2D with BMI fluctuation rate. The missing values of lifestyle behavior were imputed. Despite that, statistical significance was maintained. Also, the lack of information on the use of lipid-lowering therapy and antidiabetic and weight-loss drugs may have influenced the results. However, if this bias explained the results, then the expected change in estimates would be toward the null. The use of BMI fluctuation rate in a Cox proportional hazard analysis, which addresses the risk over time, may lead to overvaluing of our results. However, we conducted a logistic regression model after 5 years of free disease period for all the participants to exclude the possible time interaction in the Cox model, and consistent results were found. Also, the use of BMI fluctuation rate (|(kg/m^2^)/year|) could not be intuitive in clinical practice, but our data upraise the idea of the use of weight time-rated fluctuation as a stronger predictor rather than weight fluctuations. Finally, residual confounding by unmeasured factors is always a possibility in observational research, and causal relations cannot be definitely established but can be a consequence. Nevertheless, the main known risk factors for CVD in the adjustment were included, which could act as markers of a general healthier lifestyle.

In conclusion, BMI fluctuation rate (|(kg/m^2^)/year|) was associated with a significantly increased risk of CVD in this clinical cohort. The magnitude of the risk was stronger than using BMI fluctuations (|kg/m^2^|). This finding may suggest that the time-rated weight fluctuation is a more important determinant to increase the risk of CVD than the weight fluctuation itself. Overall, clinical interventions for avoiding quick weight cycling are needed to prevent future cardiovascular events related to stress comorbidities and associated complications within precision medicine strategies (28).

## Data availability statement

The raw data supporting the conclusions of this article will be made available by the authors, without undue reservation.

## Ethics statement

The studies involving human participants were reviewed and approved by Ethics Committee of the Universidad de Navarra (30/2015). Written informed consent for participation was not required for this study in accordance with the national legislation and the institutional requirements.

## Author contributions

LS-I contributed to the generation of the database, performed the data analysis, and drafted the manuscript. DN-G contributed to the generation of the database and data research, extracted data, performed the data analysis, and drafted the manuscript. DM-U contributed to the discussion and the design and intellectual revision of the manuscript. JP reviewed the data analysis and contributed to the discussion, data interpretation, and design and intellectual revision of the manuscript. AF-M conceived the plan of analysis, performed the data analysis, and contributed to the design, data analysis and interpretation, and intellectual revision of the manuscript. JM contributed to the design, data analysis and interpretation, and intellectual revision of the manuscript. All authors made substantial contributions to the conception and design and analysis and interpretation of data, drafted or revised the article critically for important intellectual content, and approved the final version to be published.
