# Supplementary material for: Repercussions of absolute and time-rated BMI “yo-yo” fluctuations on cardiovascular stress-related morbidities within the vascular-metabolic CUN cohort
Source: Front Endocrinol (Lausanne). 2023 Jan 9;13:1087554. doi: 10.3389/fendo.2022.1087554 (PMC9868691; doi:10.3389/fendo.2022.1087554)
Supplement: Supplementary file 1 [file Table_1.docx]

Supplemental table 1. Association between cardiovascular disease (CVD) according to the body mass index (BMI) fluctuation (|kg/m^2^|) and BMI fluctuation rate (|kg/m^2^/year|) of 3409 participants during 5 years of free-disease follow up drawn from the Vascular Metabolic CUN clinical cohort

|  |  |  | **BMI fluctuation and incident cardiovascular disease** | | | | | |
| --- | --- | --- | --- | --- | --- | --- | --- | --- |
|  |  |  | **Crude** | | **Age and sex adjusted** | | **Multivariate adjusted model** | |
|  | n | Incident cases | OR (95% CI) | *p* | OR (95% CI) | *p* | OR (95% CI) | *p* |
| Q1 | 853 | 52 | 1 (Ref) |  | 1 (Ref) |  | 1 (Ref) |  |
| Q2 | 852 | 60 | 1.18 (0.79-1.71) | 0.431 | 1.09 (0.74-1.60) | 0.667 | 1.24 (0.83-1.84) | 0.297 |
| Q3 | 852 | 69 | 1.36 (0.93-1.97) | 0.108 | 1.34 (0.92-1.96) | 0.124 | 1.59 (1.07-2.38) | 0.023 |
| Q4 | 852 | 76 | 1.51 (1.05-2.18) | 0.028 | 1.61 (1.11-2.32) | 0.012 | 1.95 (1.28-2.96) | 0.002 |

|  |  |  | **BMI fluctuation rate and incident cardiovascular disease** | | | | | |
| --- | --- | --- | --- | --- | --- | --- | --- | --- |
|  |  |  | **Crude** | | **Age and sex adjusted** | | **Multivariate adjusted model** | |
|  | n | Incident cases | OR (95% CI) | *p* | OR (95% CI) | *p* | OR (95% CI) | *p* |
| Q1 | 853 | 43 | 1 (Ref) |  | 1 (Ref) |  | 1 (Ref) |  |
| Q2 | 852 | 57 | 1.35 (0.90-2.03) | 0.149 | 1.29 (0.86-1.94) | 0.225 | 1.45 (0.95-2.20) | 0.087 |
| Q3 | 852 | 73 | 1.77 (1.20-2.61) | 0.004 | 1.72 (1.16-2.55) | 0.006 | 2.19 (1.44-3.32) | <0.001 |
| Q4 | 852 | 84 | 2.06 (1.41-3.01) | <0.001 | 2.04 (1.39-2.99) | <0.001 | 3.45 (2.22-5.37) | <0.001 |

^a^ The logistic regression model was adjusted for age, sex, baseline BMI, cigarette smoking (never, current and former smokers), daily alcohol intake (yes/no), lifestyle pattern (physically active/sedentary behaviour), hypertension, type 2 diabetes, antiaggregation therapy, number of visits during follow up, direction of the change in BMI (neutral/positive/negative), HDL-cholesterol, LDL-cholesterol and triglycerides.
